# Sources of Blood Lead Exposure in Rural Bangladesh

**DOI:** 10.1021/acs.est.9b00744

**Published:** 2019-09-17

**Authors:** Jenna E. Forsyth, Karrie L. Weaver, Kate Maher, M. Saiful Islam, Rubhana Raqib, Mahbubur Rahman, Scott Fendorf, Stephen P Luby

**Affiliations:** † Emmett Interdisciplinary Program in Environment and Resources; ‡ Earth System Science; ⊥ Stanford Center for Innovation in Global Health, and; # Stanford Woods Institute for the Environment, Stanford University, Stanford, California 94305, United States; § Infectious Diseases Division and; ∥ Environmental Interventions Unit, International Centre for Diarrhoeal Disease Research, Bangladesh, Dhaka 1212, Bangladesh

## Abstract

Lead (Pb) exposure is a major public health problem worldwide. Although high levels of Pb in blood in Bangladesh have been documented, the dominant Pb sources contributing to human exposure in rural Bangladesh have not been determined. Here, we first obtained blood from pregnant women from three rural Bangladeshi districts who were previously assessed by a case-control and sampling study, and we then conducted semistructured in-depth interviews to understand Pb exposure behavior and finally collected samples of the suspected Pb sources. We measured the Pb isotopic composition of both potential Pb sources and 45 blood samples in order to understand which of three sources predominate: (1) food from Pb-soldered cans, (2) turmeric, or (3) geophagous materials (clay, soil, or ash). The Pb isotope ratios of the three sources are distinct (*p* = 0.0001) and blood isotope ratios are most similar to turmeric. Elevated lead and chromium (Cr) concentrations in turmeric and a yellow pigment used in turmeric processing are consistent with reported consumption behavior that indicated turmeric as a primary contributor to blood Pb. The Pb isotopic composition analyses combined with a case-control and sampling approach provides evidence that turmeric adulterated with the yellow Pb-bearing pigment is the main Pb exposure source in these districts and illustrates the need to assess drivers and practices of turmeric adulteration, as well as the prevalence of adulteration across South Asia.

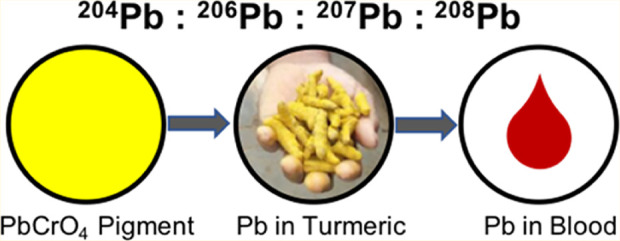

Supporting Information

## INTRODUCTION

Exposure to lead (Pb) during the prenatal period and early childhood has an irreversible negative effect on cognitive outcomes later in life.^1,2^ A pooled analysis of data from 1333 children indicated that those with blood Pb levels (BLLs) between 2.4 and 10 μ*g*/dL had subsequent IQ scores 3.9 points lower than children with BLLs < 2.4 μ*g*/dL.^3^ Additionally, it is estimated that each year there are more than 10 million disability-adjusted life years attributable to lead exposure.^4^ Based on this and other evidence, the U.S. Centers for Disease Control and Prevention established 5μ*g*/dL as the cutoff for an elevated BLL in adults and children, though no level of Pb is considered safe.^5^

In many low-and middle-income countries, Pb exposure remains high despite a worldwide reduction in BLLs due to the transition to unleaded gasoline.^6,7^ For example, Bangladesh phased out Pb in gasoline in 1999 but elevated BLLs greater than 5μ*g*/dL persist.^8−15^ Since 2012, studies of over 1200 children in three districts found that 54−78% of children have high BLLs compared to less than 3% in the United States.^10,12,16^ The reduction in IQ from these elevated BLLs in Bangladesh costs an estimated 16 billion dollars per year in lost lifetime productivity or 6% of gross domestic products.^17^

Given the high economic costs of lost productivity attributable to Pb exposure, identifying and subsequently eliminating Pb sources in the environment are important priorities.^17^ Retrospective population-based studies aim to identify Pb sources based on statistical associations between Pb in human biomarkers (blood, urine, hair) and reported behavior or geography.^10,12,14^ However, because exposure behavior is typically assessed after the exposure has occurred, suspected sources may no longer exist or no longer contain Pb.^14^ In order to evaluate sources of ongoing exposure, sampling studies assess Pb concentrations in suspected sources. However, elevated Pb concentrations in environmental sources alone do not indicate that a source is contributing to BLLs. The uptake of Pb into the body is impacted by the frequency and amount of the material being ingested, as well as the bioaccessibility of the Pb, or how readily it will be absorbed if ingested.^18^

Another approach to identifying dominant Pb sources is to measure its isotope ratios, which can serve as fingerprints linking BLLs and environmental sources.^19^ Lead isotopic analyses have revealed sources of Pb in humans, animals, and the environment.^20−27^ When there are only one or two suspected sources, binary mixing models can be applied to assess source apportionment.^19^ However, simple mixing models may not be applicable, especially when there are more than two sources^28^ or if the samples under consideration do not represent a closed system with conservative end-member mixing. Under these conditions, more complex models or multiple-lines-of-evidence, such as Pb concentrations and other contextual information, in addition to Pb isotopic compositions, may be required to elucidate likely sources.^29^

This study builds upon a previous assessment of 430 pregnant women residing in three districts of rural Bangladesh.^14^ Numerous sources were investigated including paint, water, lead acid battery recycling, soil, agrochemicals, traditional medicines, spices, rice, and other foods. Of the potential sources of Pb to the study participants, three contained Pb above the limit of detection: (1) food storage cans soldered with Pb (Figure S1), (2) turmeric, containing Pb at levels up to 100 times greater than the Bangladesh Standard Testing Institution’s limit of 2.5 μ*g/g*,^30^ and (3) geophagous materials (Figure S2), specifically fired clay tablets consumed during pregnancy (Table 1).^31,32^ The objective of this study was to identify which of these source contributes most to BLLs by assessing Pb isotopic composition, Pb concentrations, and consumption patterns. We find that individual sources are characterized by statistically significant differences among Pb isotope ratios, whereas blood Pb isotope ratios fall within a discrete range, allowing us to decipher the most likely source of Pb in prenatal blood samples.

**Table 1:** Assessment of Lead (Pb) Exposure Sources in Rural Bangladesh from a Literature Review and Sampling Assessment in the Study Region of Mymensingh, Kishoreganj, and Tangail Districts between 2015 and 2017^12,14,32,55^

exposure source^*a*^	evidence from population-and model-based studies	Pb concentration from sampling studies (median, μ*g/g*)	hypothesized ingestion mechanism	Pb inregionalsamples^*f*^
food storage cans	women with BLL^*b*^ ≥7 μ*g*/dL were 2.6 times more likely to consume from a can than women with <2 μ*g*/dL (Forsyth et al., 2018)	230 000 (Forsyth et al., 2018)	indirect oral (Pb solder from food storage can first flakes off or degrades into food before it is consumed)	yes
turmeric	turmeric hypothesized Pb source for 309 children; 78% with BLL^*b*^ ≥5 μ*g*/dL (Gleason et al., 2014)	80.0^*d*^ (Gleason et al., 2014)	direct oral (Pb-contaminated turmeric)	yes
clay	estimated intake of 210 *μ*g Pb per day from clay tablets^*c*^ (Al-Rmalli et al., 2010)	24.7 (Al-Rmalli et al., 2010)	direct oral (natural geogenic Pb in clay)	yes
agrochemicals	women with BLL^*b*^ ≥7 μ*g*/dL were 2.9−3.6 times more likely to be in households using agrochemicals than women with <2 μ*g*/dL (Forsyth et al., 2018)	<LOD^*e*^ (Forsyth et al., 2018)	indirect oral (agrochemicals transferred to food or soil then consumed advertently or inadvertently)	no
rice	estimated intake of 6.5 μ*g* Pb per day from rice^*c*^ (Bergkvist et al., 2010)	0.013 (Bergkvist et al., 2010)	direct oral (grinding stones repaired with Pb used to grind rice which is then contaminated and consumed OR consuming rice that is contaminated with Pb-containing agrochemicals)	no
	women with BLL^*b*^ ≥7 μ*g*/dL were 3.3 times more likely to be in households grinding rice than women with <2 μ*g*/dL (Forsyth et al., 2018)	<LOD^*e*^ (Forsyth et al., 2018)		
Traditionalmedicines	mean BLL^*b*^ 2.3 μ*g*/dL higher for users of traditional medicines (Mitra et al., 2012)	<LOD^*e*^ (Forsyth et al., 2018)	direct oral (Pb used as an ingredient in traditional medicines)	no

a Paint, Pb acid battery recycling, and other industrial sources were not relevant for this rural population.^14^

b Blood lead level.

c Maximum daily intake limit set at 6 *μ*g Pb per day by the U.S. Food and Drug Administration 1993.^56^

d Mean value.

e Limit of detection (0.01 *μ*g/g via ICP-MS).

f Samples taken from study participants’ districts.

## METHODS

**Study Population and Blood Sampling.** The study population was drawn from a subset of individuals^14^ nested in the water, sanitation, hygiene benefits trial (www.washbenefits.net.bd) which followed 5551 pregnant women and children from four rural districts of Bangladesh between 2012 and 2015.^33^ We obtained written informed consent from all study participants. The study protocol was reviewed and approved by the ethical review committee at icddr,b and Stanford University. Whole blood samples were collected using trace-metal free needles from 430 randomly selected pregnant women and preserved at −80 °C in trace-metal free vials until needed for analyses (described in Forsyth et al., 2018^14^).

Forty-five pregnant women were selected for inclusion in this isotope study based on district of residence, BLL, and remaining sample blood volume. These women resided in three rural districts in Bangladesh: Mymensingh, Kishoreganj, and Tangail (Figure S3).

**Environmental Sampling and Consumption Behavior Interviews**. To inform sampling and to understand consumption behavior of the suspected sources, 60 min semistructured in-depth interviews were conducted with 20 study participants (10 with high BLLs, >5 μ*g*/dL, and 10 with low BLLs, <2 μ*g*/dL) as well as 40 additional residents from the study region. Samples were collected from study participants and neighboring residents living in all three districts who exhibited knowledge about or experience with an exposure pathway. The three suspected sources, (1) food stored in Pb-soldered cans, (2) turmeric, and (3) clay, soil, or ash consumed during pregnancy, were obtained for Pb concentration and Pb isotope ratio measurements. Samples were also collected from major retail and wholesale markets serving the region. More details about exposure source sample collection and interviewee selection are provided in the Supporting Information.

Five Pb-soldered food storage cans were collected from study participants (see Forsyth et al., 2018^14^ and Table S1 which details the sampling and measurements conducted in the parent vs the current study). To assess the extent of Pb contamination from solder, samples of stored food were collected from 20 residents who owned Pb-soldered cans. As a comparison, samples of the same food types were collected from 20 residents who stored food in cans free of Pb solder. A laboratory experiment was additionally conducted to test the feasibility of Pb transfer from solder to food (see Supporting Information and Figure S4 for more details).

Research assistants visited the four major retail markets most frequented by study participants to collect every type of turmeric available for sale. Turmeric types included loose powdered turmeric sold in 20 kg burlap sacks, packaged powdered turmeric, and dried turmeric roots. Within a market, research assistants inquired with each vendor about the production and processing history of the turmeric in order to minimize duplicate sampling. If multiple vendors sold the same brand of packaged turmeric powder, only samples of the same brand were obtained if the lot number differed. Four wholesalers in the region were visited and samples of a bright-colored yellow pigment powder used in turmeric processing, locally called peuri, were collected. By tracking the distribution of turmeric processed with the pigment, additional samples of turmeric powder and root were collected.

From 20 study participants, research assistants collected each type of geophagous material (e.g., ash, soil, or clay) reported to be consumed during pregnancy. Ash samples contained a mixture of ash and soil that women scraped from an outdoor earthen stove (Figure S2). Clay samples consisted primarily of tirhi, a type of fired clay tablet specifically formulated for pregnant women and consumed like lozenges, as well as clay pots and toys that women chewed (Figure S2). Additional samples were collected from nearby vendors selling clay pots and tirhi (Figure S2).

**Sample Digestion**. All samples were acid digested according to media-specific methods. Blood samples were dissolved in concentrated HNO_3_ and heated at 90 °C overnight. Solder from food storage cans was dissolved at room temperature in concentrated HCl for 48 h. Clay and ash were dissolved in concentrated HF and HNO_3_. Turmeric and food samples were dissolved in concentrated HNO_3_ and digested via microwave digestion (MARSXpress, CEM Corporation). More details can be found in the Supporting Information Section 1.3 and in Table S2.

**Pb Concentration Measurements.** Pb concentrations of undigested blood samples were analyzed via graphite furnace atomic absorption spectrometry at the Nutritional Biochemistry Laboratory at the International Center for Diarrheal Disease Research, Bangladesh (icddr,b) following the established protocol.^34^ All source samples were analyzed for Pb concentration via inductively coupled plasma mass spectrometry (Thermo Scientific XSERIES 2 Quadrupole ICP-MS), except pigment, clay, and ash which were analyzed via X-ray fluorescence (Spectro XEPOS HE XRF, XLab Pro 5.1 software). For ICP-MS analyses, blanks and a reference standard were analyzed every 20 samples and digestion blanks were analyzed every 40 samples. Pigments were further analyzed by X-ray diffraction to identify the dominant Pb species (Rigaku mini flex 600 using a Cu source and Si strip detector). Samples of turmeric and pigment were analyzed for chromium (Cr) in addition to Pb concentrations. Detailed QA/QC information is provided in the Supporting Information, Sections 1.4 and 1.5.

**Pb Isotopic Composition Analysis.** Samples were selected for Pb isotopic analyses to include the range in Pb concentrations represented across the source types (see Tables 2 and S7). All stable masses of Pb, including the least abundant ^204^Pb, were analyzed by multicollector inductively coupled plasma mass spectrometry (Nu plasma high-resolution MC-ICP-MS) at the Stanford ICPMS/TIMS Clean lab facility. To provide the best compositional match between the analyzed solutions and the standards, Pb was isolated from samples digests using ion exchange chromatography (details of chemical separation procedure are available in the Supporting Information).

**Table 2:** Pb Concentrations (Mean, Standard Deviation, Median, Interquartile Range, Minimum, and Maximum) of Source Samples Measured by Inductively Coupled Plasma Mass Spectrometry^*a*^

sample type	number of samples	mean [Pb] μ*g/g* (SD)	median [Pb] μ*g/g* (IQR)	min [Pb]μ*g/g*	max [Pb]μ*g/g*
**Blood Samples**					
high BLL (>5 μ*g*/dL Pb)^*b*^	36	10.3 (5.5)	7.9 (6.9−10.2)	6.6	29.1
low BLL (<2 μ*g*/dL Pb)^*b*^	9	1.7 (0.3)	1.8 (1.8−1.9)	1.1	1.9
**Turmeric-Related Samples**					
loose turmeric powder (market)	21	19.0 (68.3)	1.9 (1.5−3.0)	0.3	292.3
packaged turmeric powder (market)	7	4.0 (7.1)	0.4 (0.3−2.2)	0.1	18
loose turmeric powder (pigment-processed)	8	283.9 (420.4)	67.6 (15.8−370.8)	3.5	1151.9
turmeric root (pigment-processed)	5	413.9 (364.5)	320.5 (195.9−488.4)	62.5	1002.2
yellow pigment^*c*^	3	7.8 × 10^4^ (2.0 × 10^4^)	7.2 × 10^4^	6.2 × 10^4^	1.0 × 10^5^
**Geophagous Samples**					
ash	20	33.3 (6.0)	31.9 (29.2−35.9)	27.3	50.5
clay	8	42.2 (2.4)	42.2 (40.7−43.9)	38.3	45.6
**Solder-Related Samples**					
Pb solder from food storage cans^*d*^	5	2.5 × 10^5^ (1.0 × 10^5^)	2.5 × 10^5^ (2.5 × 10^5^ to 2.8 × 10^6^)	1.0 × 10^5^	3.9 × 10^6^
food stored in Pb-soldered cans	17	2.8 (6.2)	0.2 (<LOD − 0.5)	<LOD	20.3
food stored in Pb-free cans	25	0.1 (0.2)	0.1 (<LOD − 0.1)	<LOD	0.8

a Limit of detection (LOD) was 0.001 μ*g/g*

b Blood measurements in μ*g*/dL Pb.

c Measured with X-ray fluorescence, LOD 0.2 μ*g/g* Pb.

d Measured in Forsyth et al.^14^

Isotopic compositions of blood and potential source materials were measured in triplicate. To increase sensitivity, samples were aspirated (Nu desolvation nebuliser system) and analyzed as a dry plasma. Uptake rates were 50 μ*L*/min, and sample solution concentrations were approximately 10 ng/mL measured at masses ^202^Hg, ^204^Pb, ^206^Pb, ^207^Pb, and ^208^Pb. Low BLL samples were analyzed using electron multipliers (Supporting Information Section 1.5 and Table S3), and all other samples were analyzed using Faraday cups. Both methods used a sample-standard bracketing approach with SRM 981 as the bracketing standard.^35^ External reproducibility was monitored through the analysis of the United States Geological Survey basaltic rock standard BCR-2.^36^ Detailed isotope measurement methods, including instrument parameters, can be found in the Supporting Information Section 1.5 We examined all isotope ratios and graphically present ^207^Pb/^204^Pb versus ^206^Pb/^204^Pb for discussion. ^208^Pb/^206^Pb versus ^207^Pb/^206^Pb is presented in the Supporting Information for comparison with other environmental health studies. We performed a nonparametric Kruskal−Wallis one-way analysis of variance by ranks test to assess differences in all five isotope ratios among sources (^206^Pb/^204^Pb, ^207^Pb/^204^Pb, ^208^Pb/^204^Pb, ^207^Pb/^206^Pb, and ^208^Pb/^206^Pb). We conducted post hoc Mann−Whitney U tests for pair-wise comparisons of blood isotopes with individual source isotopes.

## RESULTS AND DISCUSSION

**Pb Concentrations and Consumption Behavior.** As summarized in Table 2, Pb concentrations among the sources were highly variable. Although can solder has extremely high Pb concentrations, 10−39% Pb by weight, food stored in Pb-soldered cans has much lower concentrations with a maximum of 20.3 μ*g/g* Pb. Overall, only 3 of 17 food samples from Pb-soldered cans contain detectable Pb compared to none of the 25 samples from Pb-free containers (Tables 2 and S4). Food samples were primarily not only puffed rice but also uncooked rice, lentils, and spices. Only 23% of participants from the study region reported consuming food stored in cans.^14^ The amount of food consumed varied, though the majority reported consuming small quantities of puffed rice every day or every few days. Although we did not measure Pb bioaccessibility, a study using an in vitro bioaccessibility assay found that Pb solder particles were on average only 5% bioaccessible.^37^ Thus, even though Pb solder particles contaminate dried foods, the low Pb concentrations and limited consumption suggest that Pb from this source did not contribute to BLLs of most study participants.

Although clay and ash Pb concentrations were higher than food Pb concentrations, geophagy is unlikely to be a dominant Pb exposure route because it is uncommon among study participants. Our study found that average clay and ash Pb concentrations were 42.2 and 33.3 μ*g/g*, respectively, similar to those reported in a study of geophagy among Bangladeshi women living in the UK (Tables 2 and S5).^31^ Previous work showed that the Pb in imported clay tablets was only 0.5−4.1% bioaccessible in the gastric phase according to a physiologically based extraction test.^31^ Respondents in our study who practiced geophagy reported the allure of the “sweet earthy taste and smell” of clay and ash, consuming anywhere from a “fingerful” of ash or small pieces of clay several times a day to only once during their entire pregnancy. Either scenario would result in less than the 20−50 g of clay consumed per day reported by other studies.^31,32^ Moreover, in the present study, only 6 of 20 women reported consuming clay and ash throughout pregnancy. Although we cannot extrapolate such numbers to a population prevalence given the small sample size, but data from another study conducted in India suggest that fewer than 5% of women practice geophagy.^38^

Unlike food from Pb-soldered cans and geophagy, turmeric could be a primary Pb exposure pathway based on Pb concentrations and consumption patterns. Our results indicate that turmeric Pb concentrations were as high as 1151 μ*g/g* (Table 2). Eight of 28 market turmeric samples contained Pb above the 2.5 μ*g/g* Government of Bangladesh limit for Pb in turmeric (Table S6). Using the simplified bioaccessibility extraction test, prior studies reported that the bioaccessible fraction of Pb in turmeric varied from 42.9 to 70% of total Pb.^12,39^ Given that turmeric is used in dishes containing tamarind and other acidic ingredients, cooking could further increase the bioaccessibility of the Pb.^40^ Other researchers hypothesized that PbCrO_4_ is added to turmeric to enhance its color or weight, but they did not test any turmeric processing powders to assess molar Pb/Cr ratios or Pb speciation.^12^ We found that the yellow pigment powders used in turmeric processing contained 6−10% Pb by weight (61 870−101 300 μ*g/g* Pb). Both pigment and turmeric samples also contained elevated chromium (Cr) concentrations, with average Pb/Cr molar ratios of 1.3 ± 0.06 (2 SD) and 1.1 ± 0.8 (2 SD), respectively. X-ray diffraction analyses indicated that all three pigment samples contained lead chromate (PbCrO_4_, 10−15%), that two of the pigments also contained lead carbonate (PbCO3, 2−3%), and that one also contained lead sulfate (PbSO4, 3%). Because PbCO3 and PbSO4 have a greater bioaccessibility than PbCrO_4_, our results support the parallel findings of high turmeric bioaccessibility reported in other studies.^12,39,41^

Respondents described turmeric, primarily purchased as a loose powder, as one of three essential spices consumed daily, alongside chili powder and cumin. Women reported adding turmeric in heaping spoonfuls to curries and other dishes for at least one meal per day. Although we did not investigate the amount of turmeric being consumed, a study conducted in India reported consumption rates of 25 g of turmeric per person per month.^42^ Given the variability in turmeric Pb concentrations, the μ*g* of Pb ingested from turmeric is likely to vary most according to the distribution of turmeric adulterated with pigment. Regardless, because it is consumed daily, turmeric Pb is the most likely contributor among these sources—solder, geophagy, and turmeric—to the BLLs of the majority of women.

**Figure 1:**
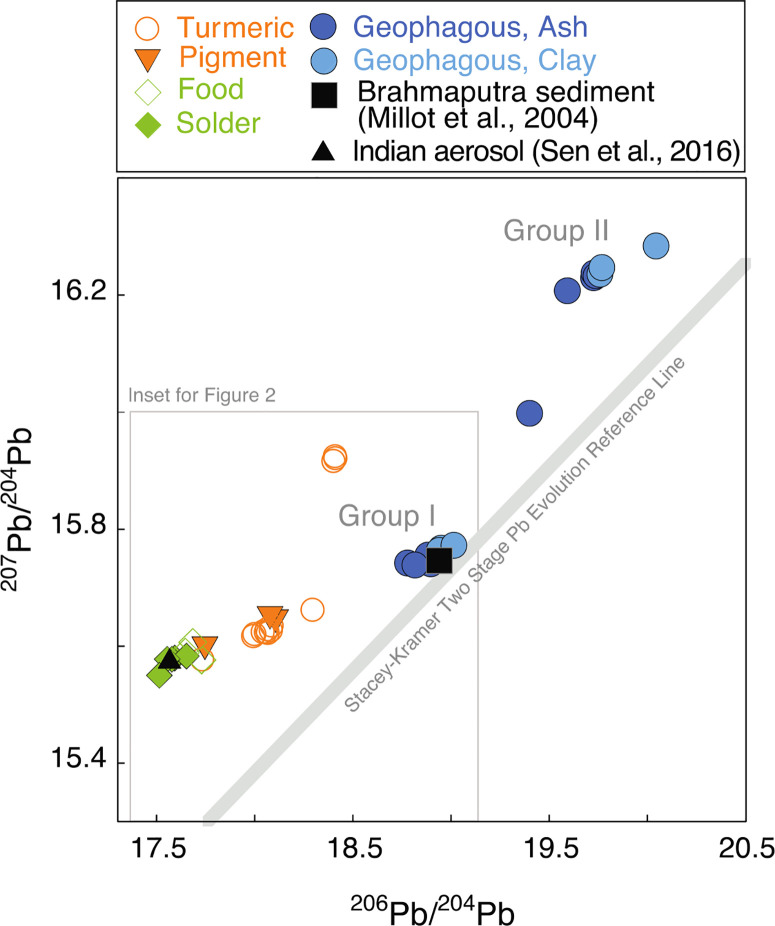
Comparison of isotope ratios (^207^Pb/^204^Pb vs ^206^Pb/^204^Pb) in Pb-soldered cans, food from Pb-soldered cans, ash, clay, turmeric, and yellow pigment collected from study participants and surrounding markets in Tangail, Mymensingh, and Kishoreganj, Bangladesh, 2015−2017. Representative reference values are shown for sediment from the Brahmaputra headwaters^45^ and for industrial aerosol from nearby Kanpur, northern India.^43^ The two-stage Pb evolution model, the 3.7 Ga terrestrial isochron, is shown for ref ^44^. Error bars are smaller than symbols (see Table S7). Inset is shown in Figure 2.

**Figure 2:**
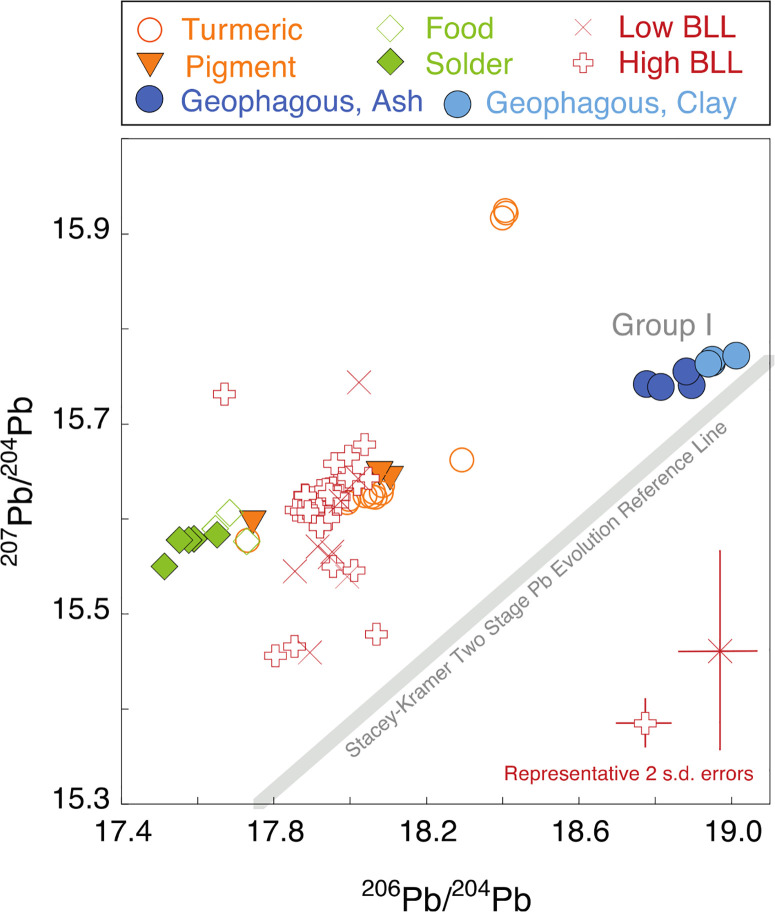
Comparison of isotope ratios (^207^Pb/^204^Pb vs ^206^Pb/^204^Pb) in women’s blood relative to Pb-soldered cans, food from Pb-soldered cans, ash, clay, turmeric, and yellow pigment collected from study participants and surrounding markets in Tangail, Mymensingh, and Kishoreganj, Bangladesh, 2015−2017. The two-stage Pb evolution line, the 3.7 Ga isochron, is shown for ref 44. Note that the scale has changed from Figure 1. Typical errors on high and low BLL samples are provided for reference (see Table S7).

**Source Pb Isotopic Compositions**. The Pb isotopic compositions of sources show a complex distribution that is best understood from the relationships between ^206^Pb/^204^Pb and ^207^Pb/^204^Pb. Overall, sources exhibit distinct fingerprints with respect to all isotope ratios (^206^Pb/^204^Pb, ^207^Pb/^204^Pb, ^208^Pb/^204^Pb, ^208^Pb/^206^Pb, and ^207^Pb/^206^Pb (*p* = 0.0001), Figures 1 and S5−S7, Table S7). We focus our discussion on the ^204^Pb-normalized data (Figures 1 and 2), which allow us to identify compositional relationships not apparent in the ^206^Pb-normalized treatment (Figure S5). Two linear trends in the ^204^Pb-normalized scatter plots (Figure 1) are inferred from the alignment with group I and group II geophagous samples and the less radiogenic industrial Pb sources. In general, group I trend has lower ^207^Pb/^204^Pb than group II materials, defined by a lower endmember of can solder, typical of industrial sources in the area.^43^ The more radiogenic compositions in group I are represented by group I geophagous samples.

Linear trends on ^204^Pb-normalized scatter plots (Figures 1 and 2) can be expected for two reasons. The first is the sampling of materials of a common geologic parent material with varying U/Pb, where the line through the individual samples defines an isochron. The second is by the mixing of materials with distinct isotopic compositions. The materials analyzed in this study are expected to be a mixture of both anthropogenic and geogenic Pb sources. For this reason, we evaluate the linear trends present in the data as potential mixing lines. For reference, we have included the Stacey− Kramers two-stage Pb evolution reference line.^44^ This line represents expected compositions of modern, average terrestrial geologic materials over a range of U/Pb compositions. It is presented here as a basis for comparison.

Geophagous samples have the most radiogenic compositions of the samples analyzed in this study. They cluster in two groups with high ^206^Pb/^204^Pb and ^207^Pb/^204^Pb values relative to samples, labeled group I, coincides with Brahmaputra river sediment which serves as a representative average of geogenic isotopic composition for the region.^45^ The Brahmaputra river starts in the Himalayas and empties into the Bay of Bengal, traveling through the study region. Four ash and three clay samples, labeled as group II, cluster at the highest ^207^Pb/^204^Pb values and may represent a different geogenic Pb reservoir in the region. One ash sample is of intermediate composition between group I and group II.

Can solder has the lowest ^206^Pb/^204^Pb and ^207^Pb/^204^Pb values, potentially on a linear trend with group I geophagous samples. A reference value for industrial aerosols from India is similar to the ^206^Pb/^204^Pb and ^207^Pb/^204^Pb isotopic composition of Pb-soldered cans and stored food from our study participants (Figure 1, Table S7).^43^ Moreover, solder and food samples have similar isotopic compositions, consistent with Pb transfer from cans to food.

Three PbCrO_4_-based pigments used in turmeric processing lie on a linear trend with isotopic compositions intermediate to the solder, food, and group I geophagous samples. One pigment has lower isotopic compositions overlapping with solder and food samples. PbCrO_4_ pigment production may involve lead oxide (PbO).^46^ Because solder also contains PbO, this could explain the similarity between isotopic compositions of the solder samples and the one pigment sample.

Turmeric samples can be grouped into two general isotopic compositions. Three of 13 samples have distinctly higher ^206^Pb/^204^Pb and ^207^Pb/^204^Pb, aligning with group II geo-phagous samples. An industrial pigment was not sampled with isotopic composition similar to these turmeric samples. However, given that these three turmeric samples contained elevated Pb concentrations ranging from 62.5 to 320.5 μ*g/g* Pb and had Pb/Cr molar ratios >1 like the pigment samples, the most probable Pb source in the turmeric is from anthropogenic pigment Pb and not from geogenic Pb from soil adhering to the turmeric roots.

The remaining 10 turmeric samples all lie on a linear trend with solder, food, pigment, and group I geophagous samples. The range of isotopic compositions of these 10 samples is completely bound by the range of isotopic compositions in the pigment. Given our Pb concentration data showing elevated Pb and Cr in both turmeric and pigments, field observations of pigments being added to turmeric roots during processing, and the coincidence among the isotopic compositions of turmeric and pigment, our results suggest that most Pb in turmeric we sampled is derived from pigments.

**Blood Pb Isotopic Compositions**. Comparing source and blood isotopic compositions, we find that turmeric adulterated with PbCrO_4_ pigments is the likely dominant contributor to blood Pb, consistent with Pb concentrations and consumption patterns. Both the ^204^Pb-normalized scatter plot (Figure 2) and the ^206^Pb-normalized scatter plot (Figure S5) reveal most overlap with turmeric values and the central tendency of the distribution of blood isotopic compositions.

Low BLL samples (<2 μ*g*/dL Pb) have the most scatter of any sample subgroup. Because the total mass of Pb available for analysis was less than 5 ng, this increased the errors on the measured isotopic compositions. In general, the low BLL samples cover a range of compositions broadly consistent with high BLL samples (>5 μ*g*/dL Pb). Blood isotopic compositions remain similar with increasing Pb concentration (Figure S8). Given the large error in low BLL samples, a more samples were below the US CDC environmental and educational intervention level of 5 μ*g*/dL and were therefore not the focus of concern. Henceforth, we focus our discussion on the high BLL samples.

High BLL samples are distinct from ash, clay, solder, and food with respect to all five isotope ratios (*P* < 0.05) but are dominantly colinear with group I source materials and overlap most with turmeric (Figures 2 and S5, Table S7). We compare the distributions of turmeric and high BLL samples using the ^204^Pb-normalized plot (Figure 1). Because the high BLL samples are more dispersed with regard to ^207^Pb/^204^Pb than ^206^Pb/^204^Pb, we identify extreme values as those that are beyond 1.5 times the interquartile range of turmeric’s ^207^Pb/^204^Pb values.^47^ One high BLL sample (12.2 μ*g*/dL Pb)has elevated ^207^Pb/^204^Pb [>(Q3_turmeric_ + 1.5IQR_turmeric_)] and appears colinear with group II turmeric and geophagous samples. Five samples exhibit low ^207^Pb/^204^Pb values [<(Q1_turmeric_ − 1.5IQR_turmeric_)], suggesting an unidentified source with an isotopic composition closer to the Stacey− Kramer reference line.^44^ Thirty-three of 39, or 85%, of high BLL samples have isotopic compositions between solder and geophagous samples and fall on the group I trend along with all solder, food, pigment, and 77% of turmeric samples. These 85% of high BLL samples are bracketed by the isotopic composition of pigment and turmeric and do not require a source other than pigment-adulterated turmeric to explain to their composition.

Although the majority of BLL samples are bracketed by the isotopic compositions of pigment and turmeric, to use the measured isotope composition and source concentration data to apportion the Pb in the blood using two or three end-member mixing models would not be appropriate in this context. Mixing models are conservative, requiring a total accounting of all of the material that enters and leaves the system (mass balance). Blood samples were collected during the second trimester of pregnancy when Pb in bones is released into the blood; thus, the measured prenatal blood Pb may be a marker of recent exposure within the past 40−60 days circulating in the blood or past Pb exposure from bone stores.^48–51^ We do not know what fraction of the Pb is from recently consumed sources versus resorption of bone Pb nor do we know the true Pb bioaccessibility from different sources. As a result, blood isotopic compositions do not represent conservative mixing of sources; thus, mass balance-based models would not be applicable. Moreover, given the collinearity of source isotopic compositions in both the ^206^Pb-and ^204^Pb-normalized spaces, and the overlap between turmeric and high BLL samples, models would not be able to quantitatively discriminate between sources.

**Limitations**. We conducted a prior case-control and sampling study in this population and ruled out water, soil, paint, lead acid battery recycling, rice, cookware, agro-chemicals, and medicines as present-day Pb sources.^14^ Thus, we have high confidence that all major Pb sources were examined. It is possible, however, that other minor Pb sources that we did not assess could contribute to the Pb of the few extreme high BLL samples with values below the group I trend. Future research could also examine other potential exposures such as atmospheric dust or biomass combustion, for example.^52–54^ However, it is clear that turmeric is the largest source of Pb within our sampling. Another limitation of this study is the small sample size that makes it difficult to generalize the measured source Pb concentrations and the reported consumption behaviors to a population prevalence. If we were to take a random sample of sources to measure Pb concentrations and bioaccessibility and survey a representative sample of the population to better understand consumption behavior, we could quantitatively model exposure. With such information, we could estimate the mass of Pb from turmeric vis-à-vis other sources and predict impacts on BLLs and IQ. Nonetheless, the Pb concentration and consumption behavior data, together with the isotopic compositions, provide evidence that support our conclusion that turmeric is the major Pb source. The isotopic compositions of 85% of high BLL samples are within 1.5 times the interquartile range of turmeric’s isotopic compositions. Turmeric contains elevated Pb concentrations because it is being adulterated with a pigment containing PbCrO_4_ and highly bioaccessible PbCO_3_ (and PbSO_4_ to a lesser extent). Moreover, study participants reported consuming turmeric daily.

**Implications for Preventing Pb Exposure.** While other studies have relied on exposure questionnaires and Pb concentration assessments alone to identify exposure sources in rural Bangladesh,^11,12^ we analyzed source consumption behavior, blood and source Pb concentrations, and Pb isotopic compositions in a population previously assessed via casecontrol and sampling methods.^14^ Our findings suggest that collecting multiple-lines-of-evidence provides clearer scientific inference that can enable better decision-making and prioritysetting for Pb abatement and control. An important area for future work is understanding the extent of turmeric adulteration with an yellow pigment, the reasons and incentives for this practice, and developing strategies to prevent it, which may include the use of food-grade, Pb-free alternatives to PbCrO4. Further efforts should expand the investigation to India and throughout the rest of South Asia where turmeric is also consumed regularly.

## ASSOCIATED CONTENT

### Supporting Information

Further details about source sample collection, isotope measurements, and additional figures and tables of results (PDF)
