# Supplementary material for: Sources of Blood Lead Exposure in Rural Bangladesh
Source: Environ Sci Technol. 2019 Sep 17;53(19):11429–36. doi: 10.1021/acs.est.9b00744 (PMC7705119; doi:10.1021/acs.est.9b00744)
Supplement: Supplementary file 1 [file EST-53-19-11429-s001.pdf]

Supporting Information

Sources of blood lead exposure in rural Bangladesh

Jenna E. Forsyth,[1*] Karrie L. Weaver,[2] Kate Maher,[2] M. Saiful Islam,[3] Rubhana Raqib,[3] Mahbubur Rahman,[4] Scott Fendorf,[2] Stephen P. Luby[5]

[1]Emmett Interdisciplinary Program in Environment and Resources, Stanford University, Stanford, California, U.S.A.

[2]Earth System Science, Stanford University, Stanford, California, U.S.A.

[3]Infectious Diseases Division, International Centre for Diarrhoeal Disease Research, Bangladesh, Dhaka, Bangladesh

[4]Environmental Interventions Unit, International Centre for Diarrhoeal Disease Research, Bangladesh, Dhaka, Bangladesh

[5]Stanford Center for Innovation in Global Health, Stanford University, Stanford, California, U.S.A.

***Corresponding author:** Jenna E. Forsyth, Emmett Interdisciplinary Program in Environment and Resources, 473 Via Ortega, Y2E2 Building, Suite 226, Stanford, CA 94305. Phone: 435-232-2955, email:jforsyth@stanford.edu

Length summary:

Pages: 21

Figures: 8

Tables: 7

TABLE OF CONTENTS

1. METHODS 3
1.1 Environmental sampling and consumption behavior interviews 3
1.2 Testing the feasibility of Pb transfer from cans to food 3
1.3 Sample digestion 4
1.4 Pb concentration measurements 4
1.5 Pb isotope measurements 5
TABLES AND FIGURES 10
Table S1 10
Table S3 10
Table S4 10
Table S5 11
Table S6 11
Table S7 12
Figure S1 14
Figure S2 14
Figure S3 15
Figure S4 16
Figure S5. 17
Figure S6a-c. 19
Figure S7a-c. 20
Figure S8 21

1. METHODS

1.1 Environmental sampling and consumption behavior interviews

Research assistants used criterion-based and snowball sampling to identify individuals with whom to conduct semi-structured interviews to learn about each source or exposure pathway and to collect samples. Criteria for selecting interviewees included a) individuals' blood Pb level (BLL) (if a study participant) and b) level of knowledge or experience about the sources of Pb. As needed, additional respondents were identified via a snowball sampling approach, whereby initial respondents were asked who else might be knowledgeable. Additional interviewees were selected by observing processing, selling, and buying behaviors of Pb-contaminated products.

To collect samples of food from Pb-soldered cans, field research assistants identified 20 individuals who consumed food from Pb-soldered cans in the study region. In order to find 20 individuals with Pb-soldered cans, research assistants screened 100 residents. Twenty more individuals were selected from the region based on similar socioeconomic status who consumed food from Pb-free containers.

To collect geophagous samples, we visited 10 study participants with high BLLs (9-29 μg/dL Pb) and 10 with < 2 μg/dL Pb. We conducted interviews to understand ingestion practices of clay and ash and to obtain samples. We conducted all interviews in Bengali, audio-recorded interviews, and later transcribed and translated them to English.

1.2 Testing the feasibility of Pb transfer from cans to food

Since all food stored in the cans was dried, the hypothesized mechanism of Pb contamination was from physical abrasion or rusting of the iron can adjacent to the Pb solder that could result in the release of solid particles of Pb entering the food. To test the mechanism and

feasibility of Pb transfer from cans into food, we conducted an experiment in duplicate with five Pb-soldered cans and one control (non-Pb-soldered) can (Figure S2). The Pb concentration of puffed rice was measured before and after 20 minutes of being shaken in these cans. Following the can experiment to elucidate the Pb transfer mechanism, puffed rice Pb concentrations ranged from 4-120 µg/g Pb compared to less than 1 µg/g Pb in the control can and less than the limit of detection (0.01 µg/g) prior to the experiment (Figure S4).

1.3 Sample digestion

Turmeric samples and food from Pb-soldered cans were digested in concentrated nitric acid (HNO_3). Solder from food storage canisters was digested in 5N hydrochloric acid (HCl). Pigments were digested with 7.5N HNO_3. Geophagous samples (ash and clay) were digested using HNO_3 and used for Pb concentration determination. For isotopic analysis, geophagous samples were prepared in a clean lab facility (details below in Pb isotope section) and digested using microwave digestion (CEM Mars Xpress) in ultra-pure concentrated HNO_3 and hydrofluoric acid (HF). Blood samples for isotopic analysis were digested with ultra-pure concentrated hydrogen peroxide (H_2O_2) and HNO_3 in a clean lab. Blood was heated on a hotplate at 90°C for 12 hours. Additional H_2O_2 was added until solution was transparent and no particles remained in solution. Table S2 provides clarification on the purity of reagents used for each type of digestion.

1.4 Pb concentration measurements

Blood Pb concentrations were measured at the Nutritional Biochemistry Laboratory at the International Center for Diarrheal Disease Research, Bangladesh (icddr,b). Samples were analyzed via atomic absorption spectroscopy following the US Centers for Disease Control and Prevention procedure for Pb in blood.[1]

For all other samples except pigment and geophagous samples, Pb concentrations of acid-digested material were analyzed by quadrupole inductively coupled plasma mass spectrometry (ICP-MS) on ThermoFisher iCap X-series in Stanford's Environmental Measurements Facility. Samples were aspirated in 2% HNO_3 in parallel with an internal standard solution to correct for instrumental drift. Samples were standardized to multi-element reference solutions. Sample Pb concentrations were reproducible to within 6% based on duplicate measurements. A sub-set of 20% of samples were analyzed for Pb concentration in duplicate. Pigment and geophagous sample Pb concentrations were measured via X-Ray Fluorescence (XRF).

1.5 Pb isotope measurements

A limited amount of blood was available for analysis in this study. With less than 25 ng total Pb, great care was taken to avoid external contamination of blood samples from naturally occurring Pb in the environment. Blood and geophagous samples were handled and prepared only in the Stanford ICPMS/TIMS Clean lab facility. The facility includes a Class <1000 clean lab with dedicated Pb workstations maintained at Class 10 conditions. All reagents were ultrapure reagents (Optima®, BDH® or Suprapur®) with less than 1 ppt (1 ng/L) of elements of interest – including Pb. All labware was acid-washed Savillex® PFA vials. All other samples – food, solder, pigment, and turmeric – were digested in a standard wet laboratory and solution aliquots were transferred to clean lab beakers for chemical separation in the clean lab. Blanks from the wet lab sample processing procedure were evaluated and determined to not contribute a significant quantity of Pb to the samples that were processed in the wet lab.

Analysis of Pb isotopes by multicollector ICP-MS (MC-ICP-MS) provides the ability to achieve higher precision than analysis of Pb isotope by single collector quadrupole ICPMS. Using simultaneous collection of all Pb masses and their isotopic ratios, the precision of the

$^{208}Pb/^{206}Pb$ can be better than 0.05%. Typical multicollector methods include the collection of the less abundant ^{204}Pb allowing more detailed examination of the isotope ratios involving ^{204}Pb and the ability to identify sources that may not be distinct on ^{206}Pb-normalized plots. For the best performance of the instrument and to provide the closest compositional match between samples and standards, Pb is separated from other elements in the sample digests. Purification and isolation of Pb was achieved through the use of anion exchange chromatographic columns (AG1x8 100-200 mesh resin). To minimize analytical blanks, blood samples were processed through small volume (100 µL) teflon columns. Samples with higher Pb concentrations were processed through larger teflon columns that contain 1 mL of resin to accommodate the larger quantity of Pb present in the sample aliquots, and to prevent contamination of the columns used for the low Pb samples. For both column sizes, samples were loaded in HBr onto pre-cleaned and conditioned anion exchange resin. Major cations were washed in HBr and Pb was collected in HCl. We followed a Pb separation technique similar to Strelow 1978, Manton 1988, Kamber and Gladu 2009, and Kraus and Nelson 1958.[2-5] Purified Pb fractions were dried and treated with concentrated HNO_3 and H_2O_2 to oxidize any organic residue from the resin, a requirement for a stable signal in the mass spectrometer. Finally, dried, purified Pb separates were dissolved in 2% HNO_3 for isotopic analysis. The mass of each sample loaded onto the columns varied with sample Pb concentration to target a total Pb mass of at least 1 µg for mass spectrometric analysis. Blood was sample limited and all of the available blood sample was used. Procedural blanks for samples dissolved and processed on the lower volume columns contained 20 pg Pb which represents less than a 1% contribution to the smallest blood samples (2 ng) and is considered negligible. Procedural blanks for the higher Pb concentration samples processed via microwave digestion and larger ion exchange columns averaged 106 pg Pb (n=6) representing less than

0.05% of the total average sample separation of greater than 1 µg. Column yields were greater than 85%.

Pb isotopic composition measurements were made using a Nu Plasma High Resolution MC-ICP-MS. The 14 Faraday and 3 ion counting detectors allow for the simultaneous determination of multiple Pb isotope ratios. Samples containing greater than 5 ng Pb were analyzed using Faraday detectors and smaller sample sizes (low BLL samples) were analyzed using the ion counting detectors.

To increase sensitivity, samples were aspirated through a Nu Instruments Desolvating Nebuliser and analyzed as a dry plasma. Uptake rates were 50 µL/min and sample solution concentrations were approximately 10 ng/mL measured at masses ^{202}Hg,^{204}Pb,^{206}Pb,^{207}Pb, and ^{208}Pb. ^{202}Hg was monitored in each analysis but not readily detected in this analytical set up averaging less than 1 mV of signal. This would result in a contribution of ^{204}Hg to the ^{204}Pb signal of less than 0.02 mV and was considered negligible on the 200 mV ^{204}Pb signal. Samples were corrected for instrumental mass fractionation using a sample-standard bracketing technique with SRM-981 as the bracketing standard and assuming exponential mass fractionation. Sample-standard bracketing is a robust analytical method widely accepted for Pb isotope analyses as used by Ewing et al., 2010[6] and Oulhote et al., 2011,[7] for example, and described by Elburg et al., 2005.[8] Data were corrected to the TIMS triple-spike SRM-981 values of Galer and Abouchami, 1998.[9] External reproducibility was monitored through the analysis of the United States Geological Survey basaltic rock standard BCR-2. This standard was selected to monitor the entire analytical process from dissolution through chemistry and mass spectrometric analysis and was processed in parallel with all sample types. Although the major element composition of a basalt is not a direct match to all of the samples in this study, it is similar in

elemental composition and degree of crystallinity to the glassy burner ash and fired clay tablets which are the samples in this study that are most difficult to dissolve. It is a good monitor of possible Pb loss to fluorides or Pb contamination during digestion. The wide range of major and trace elements in BCR-2 make it a robust monitor of the chemical separation process used with the most complex sample types in this study. A well characterized, high precision Pb-isotope blood standard is not available. Though having standards with compositions that closely match each sample compositions is ideal, Pb from all samples and standards have been purified using ion exchange chemistry to minimize potential matrix effects making them similar in composition at the time of analysis. The values (n=14) during the time of this study are $^{208}Pb/^{206}Pb = 2.0584 \pm 0.0014$ (2 s.d.), $^{207}Pb/^{206}Pb = 0.8302 \pm 0.0035$ (2 s.d.), $^{204}Pb/^{206}Pb = 0.0053 \pm 0.0001$ (2 s.d.), consistent with published standard values.[10]

Low BLL samples (<5 ng Pb in total sample) were analyzed by peak hopping using 3 mass cycles and measuring using 2 ion counters simultaneously (Table S3). Although peak hopping introduces more error, use of the highly sensitive ion counters is necessary to get a stable signal significantly greater than the background noise. Samples were aspirated through an Aridus II desolvation system at an uptake rate of 50-100 µL/min and sample concentration of 0.1 to 0.5 ppb. As with the previous analytical method, samples were corrected for instrumental mass bias by sample-standard bracketing to SRM-981. External reproducibility is determined by repeated analysis of the USGS rock standard BCR-2 (n=8). Long term average values are $^{208}Pb/^{206}Pb = 2.0609 \pm 0.0140$ (2 s.d), $^{207}Pb/^{206}Pb = 0.8343 \pm 0.0075$ (2 s.d), and $^{204}Pb/^{206}Pb = 0.0054 \pm 0.0003$ (2 s.d), consistent with the standard values.[10]

References

1. Centers for Disease Control and Prevention, Laboratory Procedure Manual. 2001.
2. Strelow, F. W., Distribution coefficients and anion exchange behavior of some elements in hydrobromic-nitric acid mixtures. *Analytical Chemistry* **1978,** *50* (9), 1359-1361.
3. Manton, W., Separation of Pb from young zircons by single-bead ion exchange. *Chemical Geology: Isotope Geoscience section* **1988,** *73* (2), 147-152.
4. Kamber, B. S.; Gladu, A. H., Comparison of Pb Purification by Anion-Exchange Resin Methods and Assessment of Long-Term Reproducibility of Th/U/Pb Ratio Measurements by Quadrupole ICP-MS. *Geostandards and Geoanalytical Research* **2009,** *33* (2), 169-181.
5. Kraus, K.; Nelson, F., Symposium on Ion Exchange and Chromatography in Analytical Chemistry. *ASTM Special Technical Publication* **1958,** (195), 27.
6. Ewing, S. A.; Christensen, J. N.; Brown, S. T.; Vancuren, R. A.; Cliff, S. S.; Depaolo, D. J., Pb Isotopes as an Indicator of the Asian Contribution to Particulate Air Pollution in Urban California. *Environmental Science & Technology* **2010,** *44* (23), 8911-8916.
7. Oulhote, Y.; Le Bot, B.; Poupon, J.; Lucas, J. P.; Mandin, C.; Etchevers, A.; Zmirou-Navier, D.; Glorennec, P., Identification of sources of lead exposure in French children by lead isotope analysis: a cross-sectional study. *Environ Health* **2011,** *10*, 75.
8. Elburg, M.; Vroon, P.; van der Wagt, B.; Tchalikian, A., Sr and Pb isotopic composition of five USGS glasses (BHVO-2G, BIR-1G, BCR-2G, TB-1G, NKT-1G). *Chemical Geology* **2005,** *223* (4), 196-207.
9. Galer, S. J. G.; Abouchami, W., Practical application of lead triple spiking for correction of instrumental mass discrimination. *Mineral. Mag. A* **1998,** *62*, 491--492.
10. Weis, D.; Kieffer, B.; Maerschalk, C.; Barling, J.; de Jong, J.; Williams, G. A.; Hanano, D.; Pretorius, W.; Mattielli, N.; Scoates, J. S.; Goolaerts, A.; Friedman, R. M.; Mahoney, J. B., High-precision isotopic characterization of USGS reference materials by TIMS and MC-ICP-MS. *Geochemistry, Geophysics, Geosystems* **2006,** *7* (8).
11. Forsyth, J. E.; Saiful Islam, M.; Parvez, S. M.; Raqib, R.; Sajjadur Rahman, M.; Marie Muehe, E.; Fendorf, S.; Luby, S. P., Prevalence of elevated blood lead levels among pregnant women and sources of lead exposure in rural Bangladesh: A case control study. *Environmental Research* **2018,** *166*, 1-9.
12. Millot, R.; Allègre, C.-J.; Gaillardet, J.; Roy, S., Lead isotopic systematics of major river sediments: a new estimate of the Pb isotopic composition of the Upper Continental Crust. *Chemical Geology* **2004,** *203* (1), 75-90.
13. Sen, I. S.; Bizimis, M.; Tripathi, S. N.; Paul, D., Lead isotopic fingerprinting of aerosols to characterize the sources of atmospheric lead in an industrial city of India. *Atmospheric Environment* **2016,** *129*, 27-33.

TABLES AND FIGURES

Table S1. Sample collection and analyses conducted in the parent[11] versus current study.

	Prior case-control study	Current study
Blood sample collection and [Pb] measurement	X	
Can sample collection and [Pb] measurement	X	
Food from cans sample collection and [Pb] measurement		X
Turmeric and pigments sample collection and [Pb] measurement		X
Clay and ash sampling and [Pb] measurement		X
All Pb isotope measurements		X

Table S2. Dissolution reagents for each source sample type.

**Sample type**	**Reagents**
Can solder	Concentrated HCl (TraceMetal® grade)
Burner ash/clay	Concentrated HF and HNO_3 (Optima® grade)
Turmeric	Concentrated HNO_3 (TraceMetal® grade)
Blood	Concentrated H_2O_2 and HNO_3 (Optima® grade)
Food	Concentrated HNO_3 (TraceMetal® grade)

Table S3. Dynamic routine for the collection of Pb on ion counters IC0 and IC1. The Nu Plasma HR has fixed collectors and in the mass range of Pb they are 1 atomic mass unit (AMU) apart. The ion counters are positioned with a fixed Faraday detector in between them. Multiple analytical cycles are required to collect both odd and even masses. The magnet mass is changed to cycle the masses onto the appropriate collectors.

	IC0	L3	IC1
Cycle 1	208	-	206
Cycle 2	207	-	
Cycle 3	206	-	204

Table S4. Summary of sample type, quantity, and lead (Pb) concentrations (µg/g) in food from Pb-soldered and non-Pb-soldered containers from 40 residents in the study region.

	Sample type	Number of samples	Number of samples >2.5 μg/g Pb	[Pb] if >2. 5 μg/g Pb
Pb-soldered can	Lentil	1	0	
	Rice[a]	17	2	15.3, 14.1
	Turmeric powder	1	1	20.3
Non Pb-soldered containers	Lentil	1	0	
	Rice[a]	18	0	
	Chili powder	2	0	
	Cake	4	0	

[a]uncooked, boiled, puffed, powdered, or flattened

Table S5. Summary of sample type, quantity, and Pb concentrations (μg/g) in clay and ash from study participants and neighboring markets.

Sample type	Number of samples	Mean± s.d. (μg/g)
Clay (*tirhi*)	3	43.9 ±1.6
Clay (Pot, Toy)	5	41.2 ±2.4
Ash (from burner)	20	33.6 ±6.0
Total	28	35.8 ±6.6

Table S6. Summary of turmeric samples from four major retail markets in two districts (all in μg/g).

Market	Number of Samples	Mean± s.d. (μg/g)	Number of samples >2.5 μg/g Pb
1	8	1.4± 0.8	0
2	7	45.9± 108.8	4
3	6	6.4± 7.9	3
4	7	1.7± 2.7	1
Total	28	13.7± 54.8	8

Table S7. Measured Pb isotope and concentration data for all samples.

Sample ID	Sample type	[Pb] (µg/dL or µg/g)[a]	^{208}Pb/^{206}Pb mean	^{208}Pb/^{206}Pb s.d.	^{207}Pb/^{206}Pb mean	^{207}Pb/^{206}Pb s.d.	^{204}Pb/^{206}Pb mean	^{204}Pb/^{206}Pb s.d.
B66901	Blood	1.8	2.0979	0.0166	0.8637	0.0031	0.0556	0.0001
B24302	Blood	1.1	2.1203	0.0067	0.8670	0.0005	0.0557	0.0004
B27201	Blood	9.6	2.1171	0.0006	0.8704	0.0001	0.0557	0.0000
B27503	Blood	9.0	2.1173	0.0012	0.8692	0.0003	0.0554	0.0001
B27808	Blood	1.8	2.1345	0.0037	0.8788	0.0010	0.0555	0.0001
B29503	Blood	6.8	2.1155	0.0010	0.8678	0.0007	0.0555	0.0000
B30302	Blood	15.3	2.1203	0.0012	0.8711	0.0003	0.0557	0.0000
B30705	Blood	29.1	2.1207	0.0012	0.8662	0.0002	0.0560	0.0000
B30806	Blood	20.0	2.1192	0.0005	0.8716	0.0005	0.0558	0.0000
B31002	Blood	9.3	2.1193	0.0012	0.8719	0.0003	0.0557	0.0001
B31403	Blood	19.4	2.1190	0.0002	0.8715	0.0000	0.0558	0.0000
B31501	Blood	25.6	2.1235	0.0010	0.8738	0.0004	0.0559	0.0000
B31801	Blood	8.4	2.1201	0.0009	0.8721	0.0002	0.0558	0.0001
B32503	Blood	25.0	2.1200	0.0037	0.8661	0.0021	0.0557	0.0003
B32801	Blood	1.9	2.1286	0.0037	0.8689	0.0010	0.0556	0.0001
B33804	Blood	6.7	2.1175	0.0002	0.8697	0.0001	0.0557	0.0000
B34401	Blood	7.5	2.1164	0.0000	0.8702	0.0000	0.0558	0.0000
B36202	Blood	6.6	2.1173	0.0000	0.8696	0.0003	0.0556	0.0000
B37001	Blood	12.9	2.1186	0.0012	0.8706	0.0003	0.0556	0.0001
B38301	Blood	7.9	2.1166	0.0002	0.8712	0.0000	0.0558	0.0000
B39208	Blood	13.6	2.1220	0.0001	0.8741	0.0002	0.0560	0.0001
B39301	Blood	6.6	2.1178	0.0004	0.8694	0.0001	0.0556	0.0000
B40304	Blood	6.9	2.1200	0.0003	0.8733	0.0001	0.0560	0.0001
B41001	Blood	12.2	2.1384	0.0007	0.8903	0.0009	0.0566	0.0006
B41401	Blood	6.8	2.1190	0.0006	0.8706	0.0002	0.0557	0.0000
B41802	Blood	6.7	2.1163	0.0035	0.8691	0.0032	0.0557	0.0002
B42003	Blood	7.9	2.1066	0.0004	0.8567	0.0007	0.0553	0.0001
B44405	Blood	10.2	2.1153	0.0028	0.8667	0.0016	0.0554	0.0001
B44501	Blood	7.2	2.1143	0.0039	0.8632	0.0023	0.0555	0.0002
B44602	Blood	7.1	2.1168	0.0004	0.8693	0.0001	0.0556	0.0001
B44704	Blood	7.4	2.1173	0.0005	0.8698	0.0001	0.0557	0.0000
B44901	Blood	7.0	2.1208	0.0012	0.8715	0.0002	0.0558	0.0000
B52101	Blood	10.0	2.1155	0.0012	0.8681	0.0003	0.0562	0.0001
B54001	Blood	6.8	2.1221	0.0002	0.8737	0.0003	0.0559	0.0000
B54902	Blood	1.8	2.1051	0.0040	0.8706	0.0004	0.0560	0.0000
B55702	Blood	6.9	2.1164	0.0012	0.8695	0.0004	0.0557	0.0000
B58003	Blood	1.8	2.1196	0.0012	0.8639	0.0002	0.0559	0.0000
B58902	Blood	7.2	2.1195	0.0019	0.8715	0.0029	0.0559	0.0001
B59904	Blood	1.9	2.1261	0.0106	0.8735	0.0022	0.0555	0.0002
B60701	Blood	6.6	2.1179	0.0021	0.8696	0.0027	0.0557	0.0002
B60906	Blood	1.9	2.1150	0.0029	0.8692	0.0013	0.0558	0.0001
B63004	Blood	9.1	2.1205	0.0040	0.8700	0.0037	0.0558	0.0003
B64602	Blood	7.6	2.1191	0.0013	0.8694	0.0001	0.0557	0.0000
B66801	Blood	6.7	2.1187	0.0003	0.8724	0.0001	0.0559	0.0000
B67602	Blood	9.2	2.1178	0.0012	0.8708	0.0003	0.0557	0.0001

B70003	Blood	1.7	2.0956	0.0040	0.8680	0.0033	0.0555	0.0001
CB30603	Clay	41.0	2.0863	0.0050	0.8319	0.0035	0.0528	0.0003
CP24302	Clay	44.6	2.0830	0.0006	0.8218	0.0001	0.0506	0.0000
CP30603	Clay	41.0	2.0865	0.0023	0.8321	0.0014	0.0528	0.0001
CT56002	Clay	39.9	2.0722	0.0006	0.8125	0.0001	0.0499	0.0000
SM-Tirhi	Clay	42.4	2.0849	0.0006	0.8219	0.0001	0.0506	0.0000
Tirhi27808	Clay	45.6	2.0835	0.0003	0.8296	0.0001	0.0526	0.0000
Tirhi30302	Clay	43.6	2.0878	0.0035	0.8323	0.0024	0.0528	0.0002
RSB30302	Ash	35.9	2.0912	0.0002	0.8330	0.0056	0.0529	0.0025
RSB30705	Ash	35.9	2.0959	0.0001	0.8384	0.0000	0.0533	0.0000
RSB30705	Ash	35.9	2.0900	0.0003	0.8344	0.0001	0.0530	0.0000
RSB31002	Ash	29.7	2.0844	0.0005	0.8247	0.0058	0.0516	0.0014
RSB31203	Ash	29.4	2.0936	0.0003	0.8366	0.0001	0.0532	0.0000
RSB31403	Ash	29.0	2.0866	0.0006	0.8231	0.0001	0.0507	0.0000
RSB31501	Ash	27.9	2.0860	0.0006	0.8225	0.0001	0.0507	0.0000
RSB31603	Ash	28.6	2.0884	0.0006	0.8273	0.0001	0.0510	0.0000
RSB25404	Ash	36.7	2.0867	0.0006	0.8228	0.0001	0.0507	0.0000
32203Bcan	Solder from can	214700.8	2.1318	0.0001	0.8857	0.0000	0.0569	0.0000
58902can	Solder from can	244850.3	2.1328	0.0001	0.8863	0.0000	0.0569	0.0000
60602can	Solder from can	107011.2	2.1285	0.0003	0.8828	0.0001	0.0567	0.0000
C32202A	Solder from can	393852.8	2.1345	0.0021	0.8875	0.0017	0.0570	0.0002
C63004	Solder from can	278698.6	2.1340	0.0045	0.8879	0.0036	0.0571	0.0004
L35food	Food from can	14.0	2.1310	0.0015	0.8835	0.0003	0.0567	0.0000
L38food	Food from can	20.3	2.1301	0.0009	0.8825	0.0002	0.0565	0.0000
L40food	Food from can	15.3	2.1258	0.0006	0.8786	0.0001	0.0564	0.0000
T-13	Turmeric	292.3	2.1080	0.0174	0.8680	0.0000	0.0556	0.0000
T-151	Turmeric	1151.9	2.1262	0.0002	0.8786	0.0000	0.0564	0.0000
T-235	Turmeric	1002.2	2.1167	0.0002	0.8653	0.0000	0.0554	0.0000
T-240	Turmeric	320.5	2.1162	0.0002	0.8649	0.0001	0.0543	0.0014
T-244	Turmeric	195.9	2.1162	0.0006	0.8651	0.0001	0.0543	0.0014
T-288	Turmeric	62.5	2.1160	0.0004	0.8651	0.0001	0.0543	0.0014
T-306	Turmeric	689.7	2.1206	0.0002	0.8680	0.0000	0.0556	0.0000
T-34	Turmeric	8.4	2.1156	0.0002	0.8643	0.0000	0.0553	0.0000
T-35	Turmeric	59.2	2.1165	0.0000	0.8653	0.0000	0.0554	0.0000
T15	Turmeric	3.4	2.1091	0.0002	0.8562	0.0000	0.0547	0.0000
T248-2017	Turmeric	488.4	2.1162	0.0002	0.8648	0.0000	0.0554	0.0000
T67305	Turmeric	264.5	2.1183	0.0028	0.8661	0.0018	0.0554	0.0001
T85	Turmeric	18.2	2.1158	0.0002	0.8645	0.0000	0.0553	0.0000
107-peuri	Yellow pigment	101300.0	2.1180	0.0002	0.8656	0.0000	0.0553	0.0000
108-peuri	Yellow pigment	72040.0	2.1279	0.0002	0.8789	0.0000	0.0564	0.0000
184-peuri	Yellow pigment	61870.0	2.1090	0.0002	0.8640	0.0000	0.0552	0.0000

[a] Blood Pb reported as μg/dL. All other samples reported as μg/g.

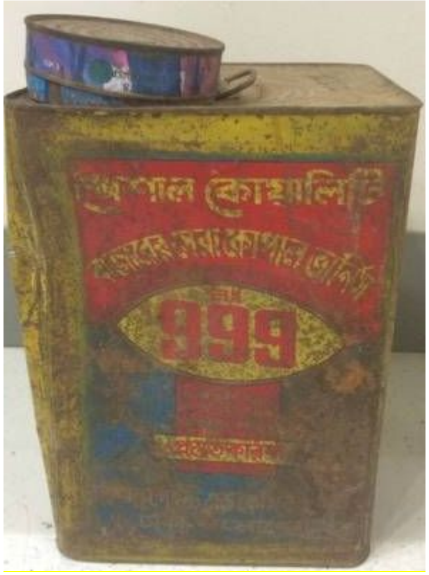

Figure S1. Pb-soldered can used to store dried foods.

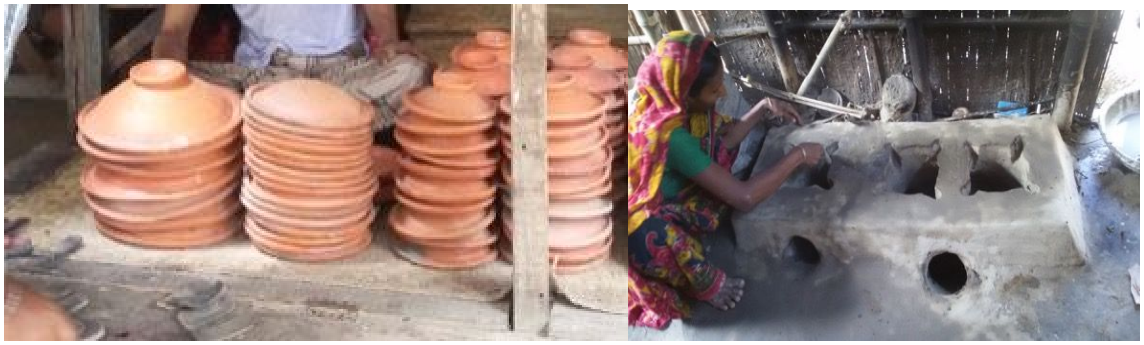

Figure S2. (Left) Clay pots in the background and clay tablets (*tirhi*) in the foreground specifically formulated and sold for pregnant women. (Right) Woman demonstrating where ash from the outdoor stove is collected for consuming during pregnancy.

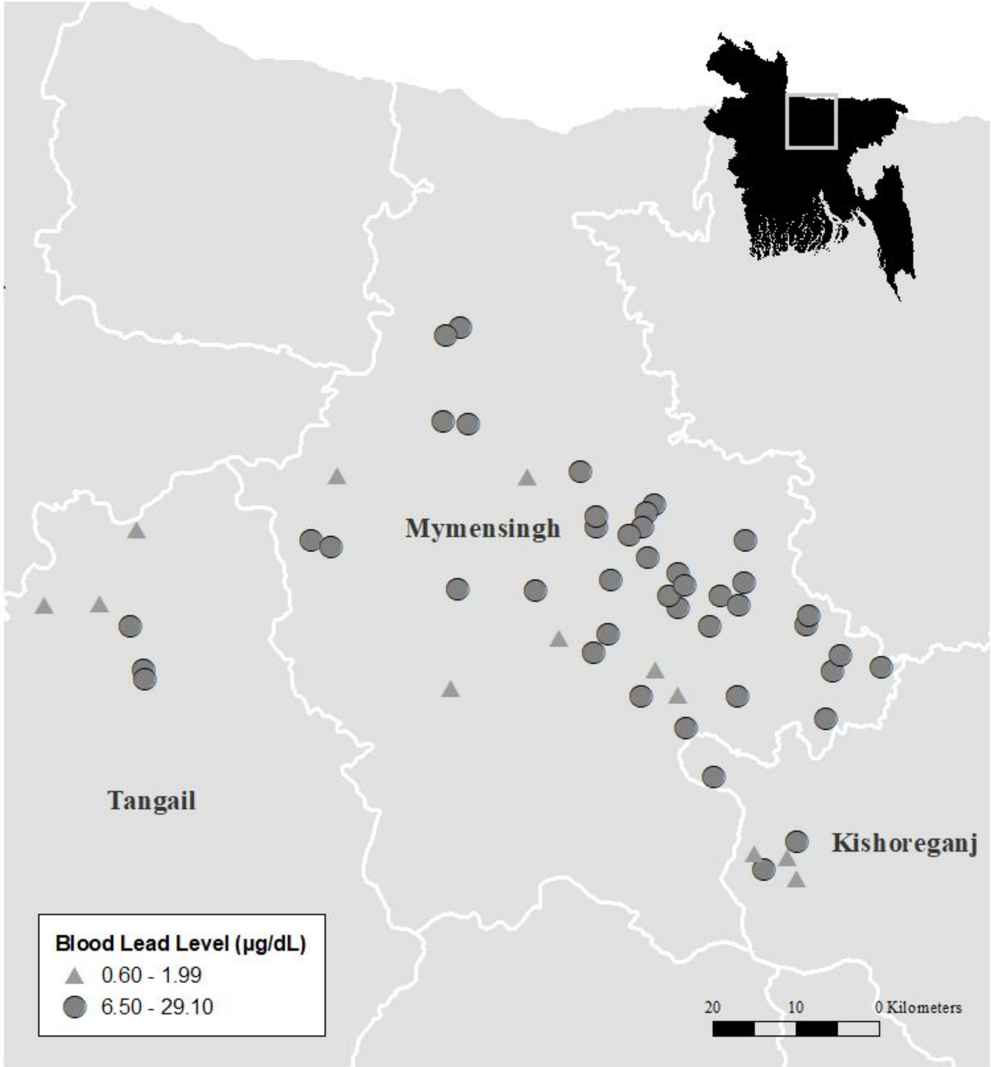

Figure S3. Location and blood lead levels (BLLs) of household participants in Tangail, Mymensingh, and Kishoreganj, three rural agrarian districts of Bangladesh. Those with elevated BLLs, >6.5 μg/dL, denoted by larger markers and household participants with low BLLs, <2 μg/dL, denoted by smaller markers.

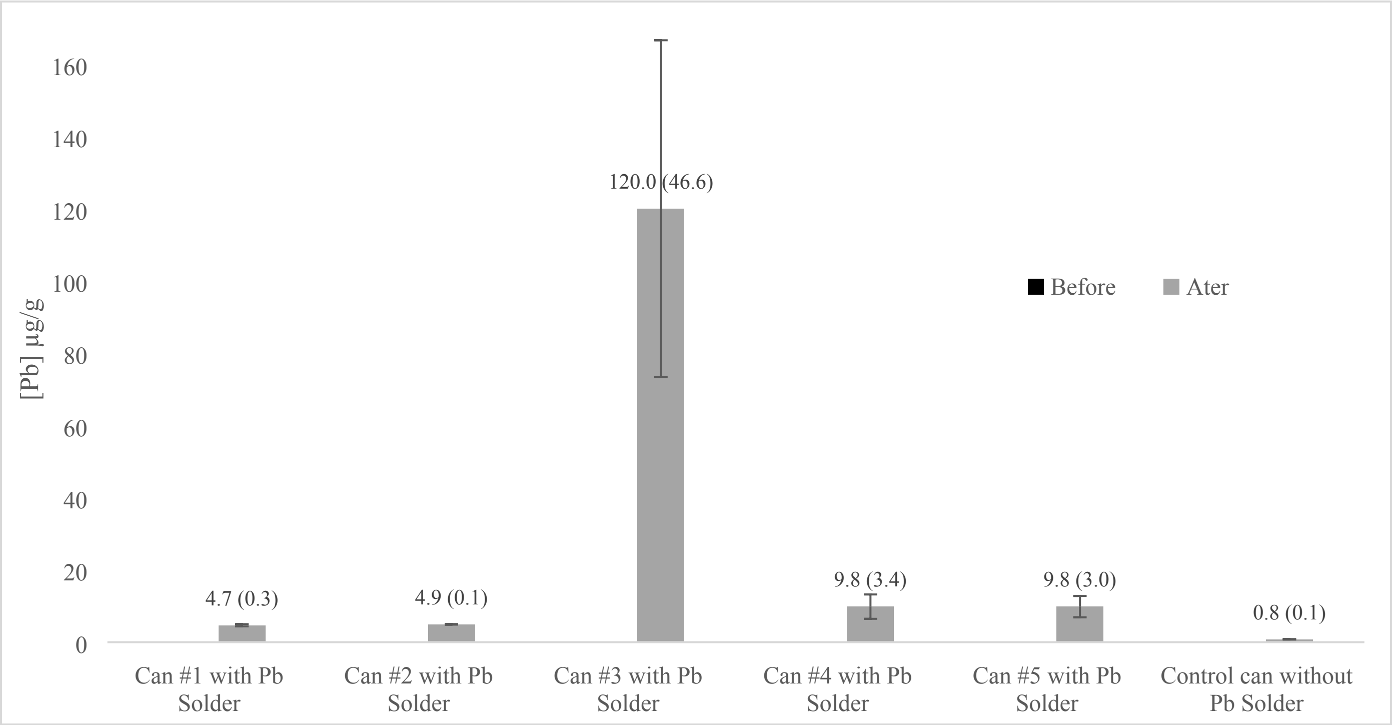

Figure S4. Mean lead (Pb) concentrations (µg/g) of puffed rice measured by ICP-MS before and after shaking in Pb-soldered cans (#1-5) and a control can with no Pb solder. Mean and standard error values from the duplicate experiment noted on the graph. Before shaking, puffed rice Pb concentrations were <LOD (0.001 µg/g).

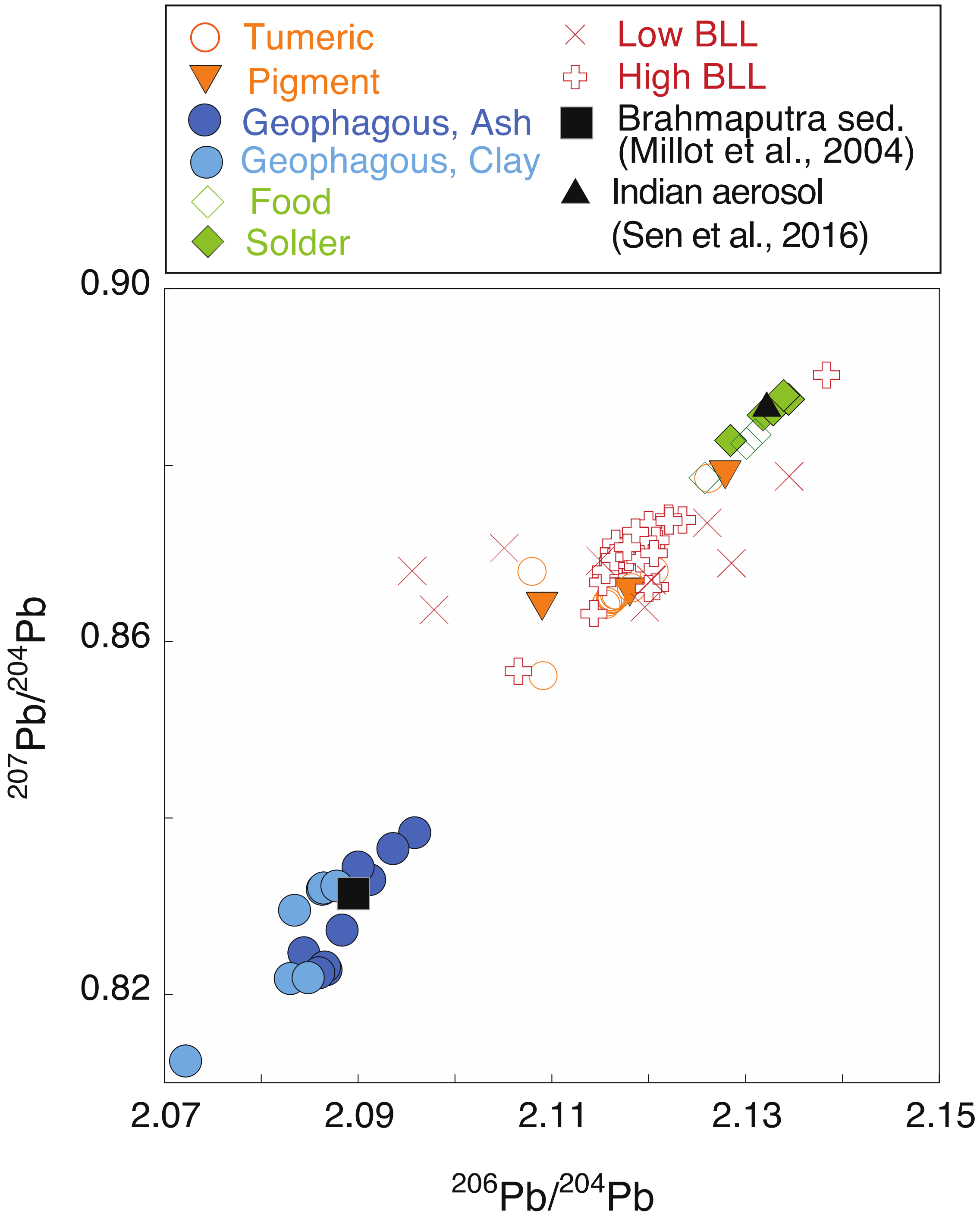

Figure S5. Comparison of isotope ratios ($^{207}Pb/^{206}Pb$ vs. $^{208}Pb/^{206}Pb$) in women's blood and Pb-soldered cans, food from Pb-soldered cans, ash, clay, turmeric, and yellow pigment collected from study participants and surrounding markets in Tangail, Mymensingh, and Kishoreganj, Bangladesh, 2015-2017. Representative reference values plotted for sediment from the nearby

region, Brahmaputra headwaters[12] and for industrial aerosols from nearby Kanpur, northern India.[13]

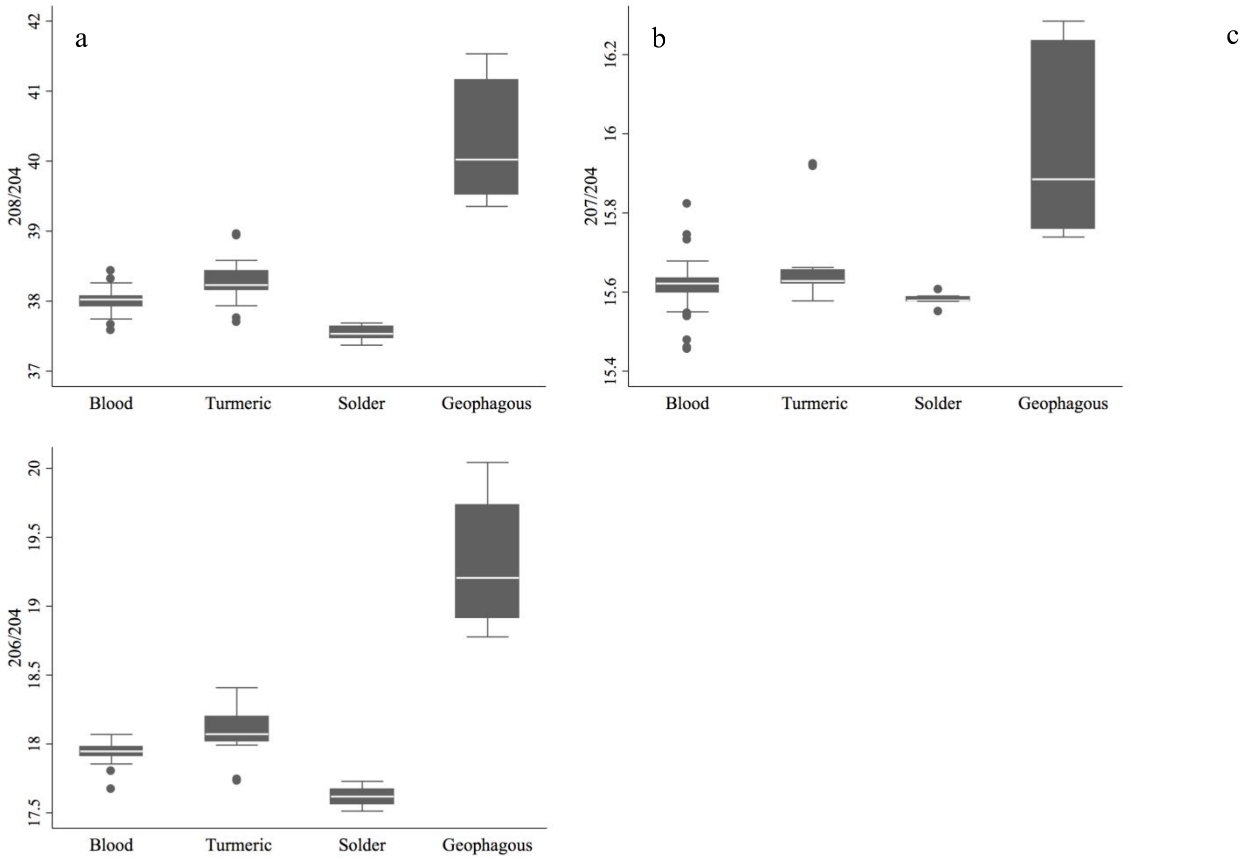

Figure S6a-c. Comparison of isotope ratios $^{208}Pb/^{204}Pb$, $^{207}Pb/^{204}Pb$, and $^{206}Pb/^{204}Pb$ for blood and Pb exposures aggregated by source type: turmeric-related materials (turmeric and yellow pigment), solder-related materials (Pb-soldered cans and food stored within), and geophagous materials (ash and clay) collected from study participants and surrounding markets in Tangail, Mymensingh, and Kishoreganj, Bangladesh, 2015-2017. Figure S6a: $^{208}Pb/^{204}Pb$ median (IQR) values for blood (38.0234 (37.9481-38.0608)), turmeric (38.2299 (38.1752-38.3612)), solder (37.5341 (37.4822-37.6206)) and ash/clay (40.0221 (39.5288-41.1600)). Figure S6b: $^{207}Pb/^{204}Pb$ median (IQR) values for blood (15.6223 (15.6036-15.6324)), turmeric (15.6278 (15.6222-15.6515)), solder (15.7849 (15.5774-

15.5849)), and ash/clay (15.8849 (15.7615-16.2339)). Figure S6c: $^{206}\text{Pb}/^{204}\text{Pb}$ median (IQR) values for blood (17.9433 (17.9069-17.9677)), turmeric (18.0711 (18.0284-18.1516)), solder (17.6181 (17.5705-17.6594)), and ash/clay (19.2051 (18.9283-19.7295)).

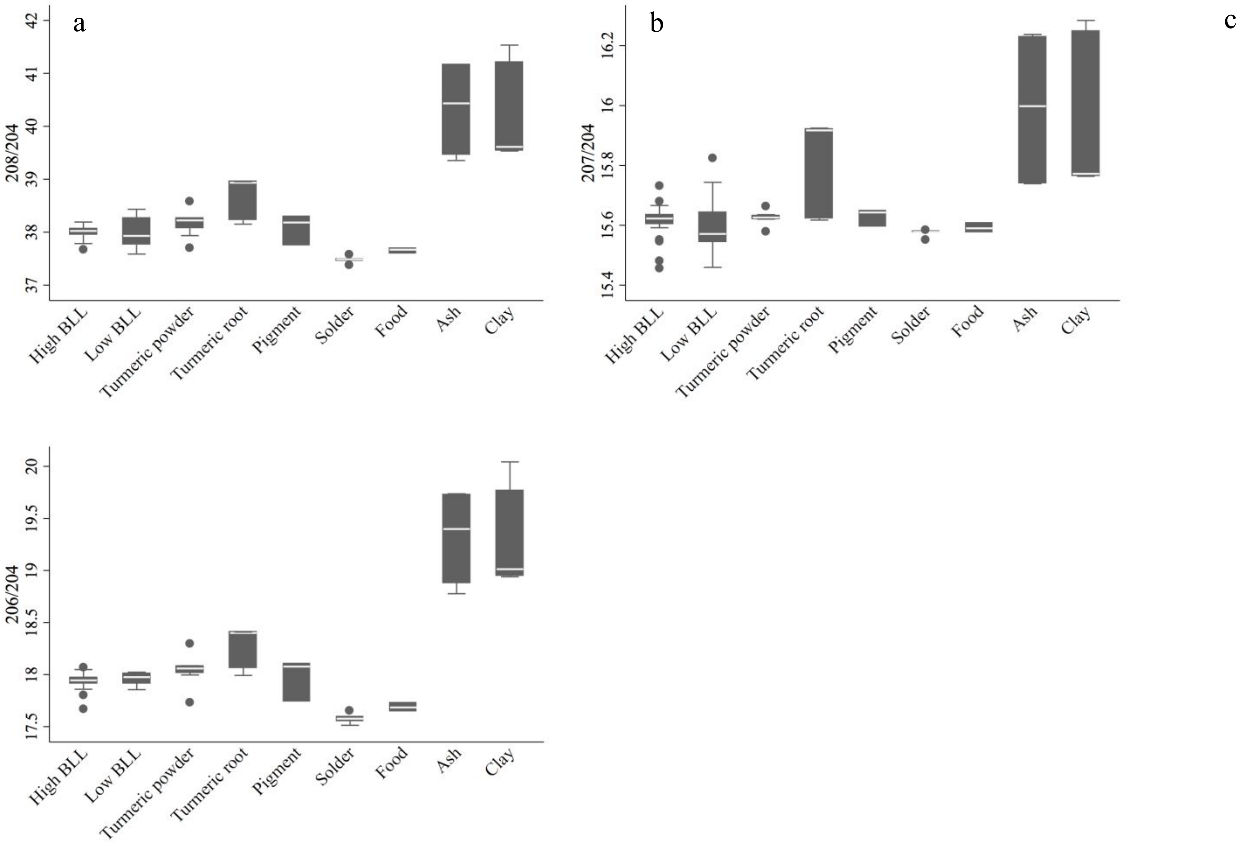

Figure S7a-c. Comparison of isotope ratios $^{208}Pb/^{204}Pb$ (Figure S7a), $^{207}Pb/^{204}Pb$ (Figure S7b), and $^{206}Pb/^{204}Pb$ (Figure S7c) for blood and Pb exposure sources disaggregated by subtypes collected from study participants and surrounding markets in Tangail, Mymensingh, and Kishoreganj, Bangladesh, 2015-2017.

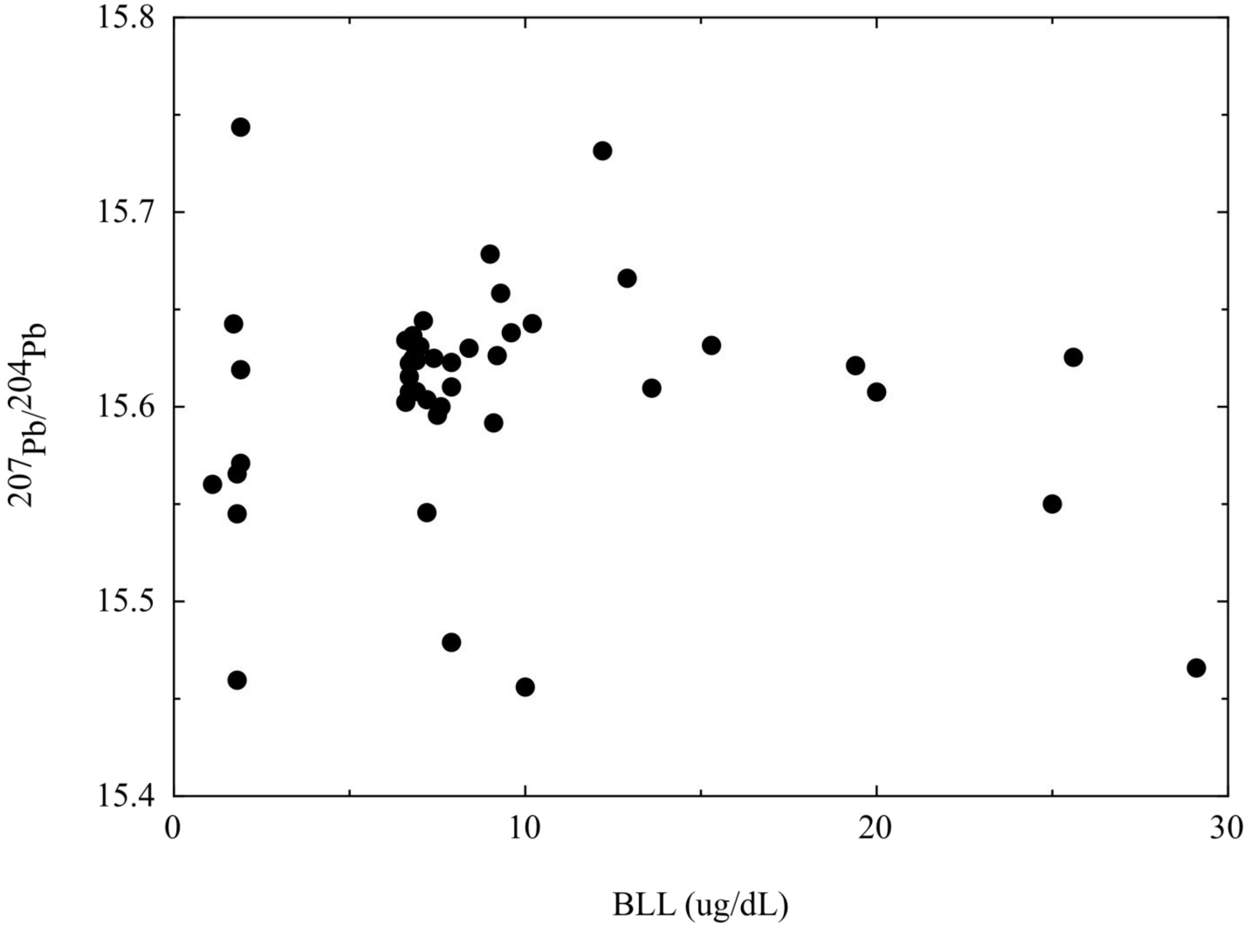

Figure S8. Isotope composition ($^{207}Pb/^{204}Pb$) vs. blood lead level (BLL, µg/dL) for forty-five female participants from rural Tangail, Mymensingh, and Kishoreganj districts, Bangladesh.
